# Vitamin D_3_ supplementation in HIV infection: effectiveness and associations with antiretroviral therapy

**DOI:** 10.1186/s12937-015-0072-6

**Published:** 2015-08-18

**Authors:** Lara Coelho, Sandra W. Cardoso, Paula M. Luz, Risa M. Hoffman, Laura Mendonça, Valdilea G. Veloso, Judith S. Currier, Beatriz Grinsztejn, Jordan E. Lake

**Affiliations:** 1 Instituto Nacional de Infectologia Evandro Chagas (INI), FIOCRUZ, Av Brasil 4365, Manguinhos, 21045-900 Rio de Janeiro, RJ Brazil; 2 University of California, Los Angeles, Los Angeles, CA USA; 3 Universidade Federal do Rio de Janeiro, Rio de Janeiro, Brazil

## Abstract

**Background:**

HIV infection and antiretroviral therapy (ART) may create unique risk factors for vitamin D insufficiency, including alterations of vitamin D metabolism by ART. We prospectively compared demographic and clinical parameters between vitamin D sufficient and insufficient HIV-infected (HIV+) adults, and assessed changes in these parameters among insufficient participants following standardized vitamin D supplementation.

**Methods:**

HIV+ adults (≥18 years old) with HIV-1 RNA <50 copies/mL on ART were enrolled. Vitamin D sufficiency and insufficiency were defined as 25-hydroxyvitamin D (25(OH)D) ≥30 or <30 ng/mL, respectively. Insufficient participants received open-label vitamin D3 50,000 IU twice weekly for 5 weeks, then 8000 IU twice weekly to complete 24 weeks. The primary endpoint was success or failure to achieve 25(OH)D ≥30 ng/mL at week 24.

**Results:**

Ninety-seven participants enrolled (34 vitamin D sufficient, 63 insufficient); 32 % female, 47 % non-White, median age 46 years, ART duration 5 years, CD4+ T lymphocyte count (CD4) 673 cells/mm^3^. 25(OH)D repletion was 83 % (95 % CI 71 %–90 %) successful. 25(OH)D levels correlated with both CD4 (*r* = 0.44, *p* = 0.01) and time on protease inhibitor (*r* = −0.35, *p* = 0.01). After adjusting for age, sex, race, nadir CD4 and baseline 25(OH)D: 1) current use of efavirenz exposure was associated with a 21.1 ng/mL higher week 24 25(OH)D level (*p* = 0.007), 2) per year use of zidovudine was associated with 7.1 ng/mL reduction in week 24 serum 25(OH)D (*p* = 0.05) and 3) every 1 ng/mL 25(OH)D increase was associated with a 3.3 cell/mm^3^ CD4 increase (*p* = 0.06).

**Conclusion:**

Vitamin D_3_ supplementation was effective in repleting 25(OH)D levels after 24 weeks. Current efavirenz use was positively associated with post-repletion 25(OH)D levels, while greater time on zidovudine was associated with lower post-repletion 25(OH)D levels. The association between improved CD4 recovery and vitamin D repletion suggests a potential benefit of vitamin D supplementation on immunologic recovery during HIV treatment.

**Trial registration:**

This trial is registered at The Brazilian Clinical Trials Registry (U1111‐1165‐2537).

**Electronic supplementary material:**

The online version of this article (doi:10.1186/s12937-015-0072-6) contains supplementary material, which is available to authorized users.

## Introduction

Low serum 25-hydroxyvitamin D levels (25(OH)D <30 ng/mL) are widely documented in both HIV-infected persons and the general population [1–5]. While serum 25(OH)D levels have been associated with seasonal variations in exposure to sunlight, high rates of vitamin D insufficiency have also been documented in regions with low latitude and year-round sun exposure, including South America [6–10].

HIV infection may create unique risk factors for vitamin D insufficiency such as chronic inflammation [11], and both the protease inhibitor (PI) and non-nucleoside reverse transcriptase inhibitor (NNRTI) classes of antiretroviral agents may enhance vitamin D metabolism via modulation of the cytochrome P450 system and vitamin D hydroxylation [1, 11–14]. Additionally, in HIV-infected persons, vitamin D insufficiency has been associated with lower CD4+ T lymphocyte counts [15, 16].

In the general population, the safety and efficacy of vitamin D supplementation has been demonstrated at a wide range of doses [17, 18]. In HIV-infected persons, the safety of vitamin D supplementation has also been described, but repletion success rates have been less consistent [3, 19]. Given the associations of vitamin D insufficiency with cardiovascular disease, insulin resistance, progression to AIDS and increased mortality [2, 15, 20–24], we designed an open-label trial of standardized vitamin D_3_ supplementation among HIV-infected men and women on suppressive combination antiretroviral therapy (ART) in Rio de Janeiro, Brazil.

## Methods

### Study design and study population

Participants were recruited from the adult HIV clinic at the Evandro Chagas Clinical Research Institute (IPEC), Oswaldo Cruz Foundation, which was established as a referral center for HIV research and care in Rio de Janeiro, Brazil in 1986. Eligible participants underwent screening for vitamin D insufficiency as part of routine clinical care between January 2011 and December 2013.

Inclusion criteria for this analysis included receipt of ART (defined as two nucleoside reverse transcriptase inhibitors (NRTI) in combination with at least one PI or one NNRTI) for at least six months prior to entry and HIV-1 RNA <50 copies/mL at study entry. ART switches in the six months prior to study enrollment were allowed for tolerability but not virologic failure. Persons receiving vitamin D supplementation >400 International Units (IU, the amount in a standard multivitamin) at screening were excluded from participation.

At screening, participants underwent serum 25(OH)D measurement via chemiluminescence assay according to the manufacturer instructions (Abbott, Chicago, IL). Vitamin D sufficiency and insufficiency were defined as 25(OH)D levels ≥30 ng/mL and <30 ng/mL, respectively. Insufficient participants were eligible to receive vitamin D_3_ (cholecalciferol) supplementation and follow-up every twelve weeks for at least 24 weeks, while those with 25(OH)D ≥30 ng/mL served as a baseline control group and did not require additional follow-up. For insufficient subjects, vitamin D_3_ supplementation adherence and tolerability were addressed at each follow-up visit. Dual-energy X-ray absorptiometry (DXA) was offered to all patients at baseline and was performed following manufacturer-recommended calibration and maintenance procedures (Lunar Prodigy densitometer, General Electric, Madison, WI). Lumbar spine and femoral neck Z scores from DXA results were used to evaluate the presence of osteopenia (Z-score between −1.0 and −2.5) or osteoporosis (Z-score ≤ −2.5).

The study protocol was approved by the Evandro Chagas Clinical Research Institute institutional review board, and all participants provided written informed consent prior to the initiation of study procedures. This trial is registered at The Brazilian Clinical Trials Registry (UTN: U1111‐1165‐2537).

### Vitamin D supplementation

Participants with insufficient serum 25(OH)D levels were prescribed a vitamin D_3_ supplementation regimen of 50,000 IU orally twice weekly for five weeks (repletion phase) followed by 8000 IU twice a week for an additional 19 weeks (maintenance phase), a regimen similar to that associated with high safety and repletion success [5, 25, 26] (see Statistical Analysis, below) and within the guidelines suggested by The Endocrine Society [27]. Vitamin D_3_ supplements were provided to participants by the IPEC research pharmacy in an emulsified 4000 IU D_3_ per drop formulation (manufacturer Roche, Basel, Switzerland). All participants received dosing and storage instructions. Adherence was measured by self-report, as pill counts could not be obtained using the emulsified D_3_ formulation.

### Data collection

Socio-demographic and clinical information were obtained from the clinical records of patients receiving HIV care at IPEC, which is updated regularly using outpatient and inpatient clinical documentation and laboratory testing results. ART use (drug, dates of use, and dose) and other data were extracted from the clinical record by trained abstractors, who recorded the information onto standardized case report forms prior to database entry.

Fasting glucose, lipid profile and glycosylated hemoglobin, and CD4+ T lymphocyte counts and HIV-1 RNA were assessed at baseline and week 24, according to local standards.

### Statistical analysis

The sample size calculation was based on the 85 % repletion success rate of a similar cholecalciferol supplementation regimen after twelve weeks in HIV-uninfected adults with low bone mineral density [5]. For this study, we predicted that HIV-infected persons might experience a less effective response to the intervention of 60 %. Based on this assumption, a minimum inclusion of 24 vitamin D insufficient participants provided 80 % power (two-tailed, α=0.05) to reject the null hypothesis that 60 % effectiveness is not statistically different from the 85 % expected repletion success rate.

The primary endpoint was dichotomized success or failure to achieve 25(OH)D ≥30 ng/mL after 24 weeks of vitamin D_3_ supplementation. Secondary endpoints included determination of associations between socio-demographic and clinical features and both baseline and week 24 25(OH)D levels, as well as an evaluation of the effects of vitamin D_3_ supplementation on metabolic and immunological parameters.

Between-group comparisons for 1) baseline vitamin D sufficiency vs. insufficiency and 2) failure vs. success of repletion after 24 weeks were performed using the Wilcoxon rank-sum test for continuous variables and the chi-square or Fisher’s exact test for categorical variables. Linear regression models were used to quantify the association of 25(OH)D levels with current ART exposure and CD4+ T lymphocyte counts. The initial multivariate models included all variables explored in the bivariate analysis. Sex, age, race and nadir CD4+ T lymphocyte count were maintained in the final multivariate models irrespective of p-values due to prior reports of associations with 25(OH)D levels. In addition, current antiretroviral drugs/classes were initially included and removed by backwards elimination until a *p*-value of < 0.05 was present for all ART variables.

## Results

### Study population

**Fig. 1:**
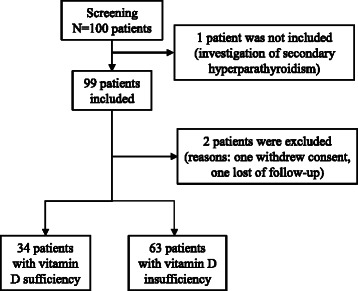
Study flow-chart: screening and enrollment of study participants

One hundred participants were screened and 99 enrolled. Of those 99, one participant withdrew consent and one was lost to follow-up, leaving 97 evaluable participants. Sixty-three participants had vitamin D insufficiency and initiated supplementation, whereas 34 participants were classified as vitamin D sufficient at baseline and were included as controls (Fig. 1).

Overall, the median age of the study population was 45 years, 68 % were men, median baseline CD4+ T lymphocyte count was 673 cells/mm^3^, median time on ART was 5 years and the most common ART agents in current use were tenofovir (71 %) and efavirenz (70 %) (Table 1).

**Table 1:** Baseline demographic and clinical characteristics

	Sufficient group	Insufficient group	Total	*P*-value
	*n* = 34	*n* = 63	*n* = 97	
25(OH)D (ng/mL)	38 (34, 43)	21 (16, 24)	24 (18, 35)	<0.001
Male sex	74 % (25)	65 % (41)	68 % (66)	0.53
Age (years)	45 (38, 50)	47 (38, 53)	45 (38, 52)	0.87
White race/ethnicity	50 % (17)	54 % (34)	53 % (51)	0.87
Nadir CD4+ T lymphocyte (cells/mm^3^)	185 (97, 230)	143 (49, 242)	170 (71, 236)	0.39
≥ 350	3 % (1)	3 % (2)	3 % (3)	
200–349	38 % (12)	34 % (21)	36 % (33)	
50–199	53 % (17)	36 % (22)	42 % (39)	
< 50	6 % (2)	26 % (16)	19 % (18)	
Baseline CD4+ T lymphocyte (cells/mm^3^)	650 (437, 754)	688 (499, 792)	673 (465, 775)	0.39
≥ 500	66 % (19)	74 % (43)	72 % (62)	
350–499	24 % (7)	16 % (9)	18 % (16)	
< 350	10 % (3)	10 % (6)	10 % (9)	
Time on ART (years)	5 (4, 5)	5 (5, 6)	5 (4, 6)	0.40
Time on TDF (years)	3 (1, 4)	3 (0, 5)	3 (1, 5)	0.42
Time on AZT (years)	0 (0, 4)	0 (0, 5)	0 (0, 5) 4(1.3,4.9)	0.06
Time on EFV (years)	3 (0, 5)	4 (2, 5)	4 (1, 5)	0.10
Time on PI (years)	2 (0, 5)	0 (0, 2)	0 (0, 5)	0.10
Current TDF use	77 % (26)	68 % (43)	71 % (69)	0.54
Current AZT use	24 % (8)	35 % (22)	31 % (30)	0.35
Current EFV use	65 % (22)	73 % (46)	70 % (68)	0.54
Current PI use	35 % (12)	24 % (15)	28 % (27)	0.33
DXA classification^a^				0.17
Normal	32 % (6)	29 % (10)	30 % (16)	
Osteopenia	63 % (12)	46 % (16)	52 % (28)	
Osteoporosis	5 % (1)	26 % (9)	18 % (10)	
Any bone disease^b^	68 % (13)	71 % (25)	70 % (38)	0.10
BMI^a^ (kg/m^2^)	25 (23, 27)	25 (24, 28)	25 (23, 27)	0.46
< 18.5	5 % (1)	0 (0)	2 % (1)	
18.5–24.9	47 % (9)	51 % (18)	50 % (27)	
25.0–29.9	47 % (9)	31 % (11)	11 % (6)	
≥ 30.0	0 (0)	17 % (6)	37 % (20)	
C-reactive protein (mg/dL)	0.2 (0.3, 0.9)	0.3 (0.2, 0.6)	0.3 (0.2, 0.6)	0.56
Glucose (mg/dL)	89 (81, 95)	88 (81, 93)	88 (81, 93)	0.73
HbA1c (%)	5.5 (5.3, 5.7)	5.5 (5.3, 5.7)	5.5 (5.3, 5.7)	0.84
Triglycerides (mg/dL)	108 (76, 181)	121 (86, 185)	120 (80, 184)	0.55
Total cholesterol (mg/dL)	195 (171, 223)	196 (173, 217)	196 (172, 221)	0.82
HDL (mg/dL)	45 (37, 51)	47 (39, 58)	47 (39, 58)	0.60
LDL (mg/dL)	117 (93, 140)	120 (92, 145)	118 (92, 143)	0.89

Median (interquartile range) or percent (n) presented

*25(OH)D* 25-hydroxyvitamin D, *TDF* tenofovir, *AZT* zidovudine, *EFV* efavirenz, *PI* protease inhibitor, *DXA* dual-energy X-ray absorptiometry, *BMI* body mass index, *HbA1c* glycated hemoglobin

^a^Data available for 54 patients (19 in sufficiency group, 35 in insufficiency group)

^b^Any bone disease aggregates osteoporosis and osteopenia

### Baseline differences by serum 25(OH)D level

At baseline, vitamin D sufficient and insufficient participants differed mainly by serum 25(OH)D level (Table 1). Although absolute current and nadir CD4+ T lymphocyte counts did not differ significantly between groups, the proportion of participants with a CD4+ T lymphocyte nadir <50 cells/mm^3^ was 26 % among insufficient participants vs. 6 % among sufficient participants (*p* = 0.04).

Regarding ART use, efavirenz use was more frequent among insufficient participants (73 % vs. 65 %, *p* = 0.54), who also had a longer exposure to this drug (*p* = 0.10). The proportion of patients on zidovudine was also higher in the insufficient group (35 % vs. 24 %, *p* = 0.35), while tenofovir and PI use were more common in the sufficient group (tenofovir: 77 % vs. 68 %, *p* = 0.54; PI: 35 % vs. 24 %, *p* = 0.33). Regarding body mass index, overall there was no significant difference between the groups, although obesity was more prevalent in the insufficient group (17 % vs. 0 %, *p* = 0.08) (Table 1).

### Baseline associations between 25(OH)D level and clinical parameters

Associations between current antiretroviral drug use and baseline 25(OH)D levels were evaluated using linear regression. In unadjusted models, per year PI exposure was associated with 1.69 ng/mL lower baseline 25(OH)D level (*p* = 0.03). Negative but non-significant associations were also observed for per year use of zidovudine, efavirenz and tenofovir. After controlling for sex, age, race and nadir CD4+ T lymphocyte count, per year exposure to any of the included ART drugs/classes was associated with lower baseline 25(OH)D levels, but those associations were non significant. Current PI use was associated with a 6.9 ng/mL higher baseline 25(OH)D level (*p* = 0.02) (Table 2A).

**Table 2:** Linear models for 25(OH)D levels (ng/mL) at baseline and at week 24 for responders (*n* = 52)

Part A.		
	Baseline (*n* = 97)	
	Unadjusted model	Adjusted model
	Beta (95 % CI)	Beta (95 % CI)
Female sex	−1.08 (−7.27, 5.12)	−5.57 (−11.40, 0.26)
Age (years)	−0.16 (−0.44, 0.11)	−0.20 (−0.46, 0.05)
Non-white race/ethnicity	2.48 (−3.29, 8.25)	2.64 (−2.78, 8.06)
Nadir CD4+ T lymphocyte (per 100 cells/mm^3^)	0.57 (−2.04, 3.18)	1.17 (−1.42, 3.75)
Current use of TDF	5.84 (−0.43, 12.12)	
Current use of AZT	−5.27 (−11.44, 0.89)	
Current use of EFV	−1.65 (−7.96, 4.66)	
Current use of PI	−2.85 (−3.55, 9.30)	**6.93 (0.96, 12.90)**
Part B.		
	Week 24^a^ (*n* = 52)	
	Unadjusted model	Adjusted model
	Beta (95 % CI)	Beta (95 % CI)
Female sex	−4.08 (−16.61, 8.46)	−0.96 (−14.20, 12.27)
Age (years)	−0.22 (−0.78, 0.33)	0.21 (−0.38, 0.79)
Non-white race/ethnicity	−5.02 (−16.95, 6.91)	−5.53 (−17.82, 6.77)
Nadir CD4+ T lymphocyte (per 100 cells/mm^3^)	1.94 (−3.44, 7.31)	3.29 (−1.96, 8.54)
Baseline 25(OH)D	**1.17 (0.25, 2.09**)	**1.27 (0.24, 2.30)**
Current use of TDF	−10.24 (−22.50, 2.02)	
Current use of AZT	7.44 (−4.70, 19.57)	
Current use of EFV	**17.88 (4.08, 31.68)**	**21.09 (6.41, 35.78)**
Current use of PI	**−16.15 (−31.10, −2.13)**	

*25(OH)D* 25-hydroxyvitamin D, *TDF* tenofovir, *AZT* zidovudine, *EFV* efavirenz, *PI* protease inhibitor

^a^For those who underwent vitamin D supplementation and achieved 25(OH)D levels > 30 ng/mL at week 24

Bold font implies statistically significant results assuming a 5% significance threshold

### Baseline associations between DXA results and demographic and clinical parameters

In the subset of participants with DXA results (*n* = 54), the prevalence of bone disease was high, with 68 % of vitamin D sufficient and 71 % of vitamin D insufficient participants having osteopenia or osteoporosis. While not statistically significant, the prevalence of osteoporosis was numerically greater in the vitamin D insufficient group (26 % vs. 5 %, *p* = 0.08) (Table 1).

25(OH)D levels did not differ between participants with any bone disease (osteoporosis or osteopenia) and those with normal Z score range (24 vs. 27 ng/mL respectively, *p* = 0.90, Additional file 1: Table S1). Participants with bone disease were more likely to be female (34 % vs. 19 %, *p* = 0.34), non-White (58 % vs. 38 %, *p* = 0.28), have a nadir CD4+ T lymphocyte count <50 cells/mm^3^ (25 % vs. 0 %, *p* = 0.04) and use efavirenz (79 % vs. 63 %, *p* = 0.31). Neither bivariate nor multivariate modeling revealed significant associations between demographic and clinical features and the presence of bone disease (data not shown).

### Vitamin D repletion effectiveness

Twenty-four weeks of vitamin D_3_ supplementation was successful in achieving serum 25(OH)D levels ≥30 ng/mL in 83 % (*n* = 52/63, 95 % CI 71–90 %) of participants. Participants were classified at week 24 as either repletion responders (25(OH)D ≥30 ng/mL) or non-responders (25(OH)D persistently <30 ng/mL), with a median post-repletion serum 25(OH)D level of 47 ng/mL among responders vs. 26 ng/mL among non-responders (Table 3).

**Table 3:** Demographic and clinical characteristics at week 24 among participants who underwent vitamin D_3_ supplementation

	WNon-responders	Responders	*P*-value
	(25(OH)D < 30 ng/mL)	(25(OH)D ≥30 ng/mL)	
	(*n* = 8)	(*n* = 52)	
25(OH)D (ng/mL)^a^	26 (25, 27)	47 (40, 65)	<0.001
Male sex	63 % (5)	65 % (34)	1.00
Age (years)	42 (36, 48)	48 (39, 54)	0.27
White race/ethnicity	63 % (5)	54 % (28)	0.72
Baseline CD4+ T lymphocyte (cells/mm^3^)	540 (509, 758)	689 (527, 833)	0.69
≥500	71 % (5)	77 % (37)	
350–499	14 % (1)	17 % (8)	
<350	14 % (1)	6 % (3)	
Week 24 CD4+ T lymphocyte (cells/mm^3^)	656 (600, 891)	712 (460, 853)	0.50
≥500	100 % (5)	67 % (22)	
350–499	0 (0)	21 % (7)	
<350	0 (0)	12 % (4)	
Time on cART (years)	5 (4, 8)	5 (5, 6)	0.96
Time on TDF (years)	4 (3, 5)	3 (0, 5)	0.30
Time on AZT (years)	0 (0, 2)	1 (0, 5)	0.50
Time on EFV (years)	2 (2, 4)	4 (3, 5)	0.31
Time on PI (years)	1 (0, 6)	0 (0, 2)	0.36
Current TDF use	100 % (8)	65 % (34)	0.09
Current AZT use	0 % (0)	39 % (20)	0.04
Current EFV use	50 % (4)	79 % (41)	0.10
Current PI use	38 % (3)	19 % (10)	0.35
Baseline BMI^b^ (kg/m^2^)	23 (23, 24)	25 (24, 28)	0.23
<18.5	0 % (0)	0 % (0)	
18.5–24.9	100 % (3)	48 % (15)	
25.0–29.9	0 % (0)	32 % (10)	
≥30.0	0 % (0)	19 % (6)	
C-reactive protein (mg/dL)	0.3 (0.2, 0.4)	0.3 (0.2, 0.9)	0.36
Glucose (mg/dL)	82 (80, 89)	91 (87, 103)	0.02
HbA1c (%)	5.5 (5.5, 5.8)	5.5 (5.2, 5.8)	0.62
Triglycerides (mg/dL)	111 (105, 128)	150 (107, 201)	0.12
Total cholesterol (mg/dL)	187 (166, 200)	198 (173, 225)	0.41
HDL (mg/dL)	51 (48, 53)	47 (40, 60)	0.52
LDL (mg/dL)	111 (94, 124)	110 (93, 133)	0.87

Median (interquartile range) or percent (n) presented

*25(OH)D* 25-hydroxyvitamin D, *TDF* tenofovir, *AZT* zidovudine, *EFV* efavirenz, *PI* protease inhibitors, *BMI* body mass index, *HbA1c* glycated hemoglobin

^a^No available 25(OH)D at week 24 for 3 participants

^b^Data available for 54 patients (19 in sufficiency group, 35 in deficiency group)

Overall, non-responders were somewhat younger (median 42 vs. 48 years, *p* = 0.27), had lower nadir and baseline CD4+ T lymphocyte counts (baseline median 540 vs. 689 cells/mm^3^, *p* = 0.69), and were more likely to use tenofovir (100 % vs. 65 %, *p* = 0.09) and PIs than responders (38 % vs. 19 %, *p* = 0.35). Non-responders also had a significantly lower proportion of current zidovudine use (0 % vs. 39 %, *p* = 0.04) and higher post-repletion fasting glucose levels (91 vs. 82 mg/dL, *p* = 0.02) (Table 3).

### Associations between successful vitamin D repletion and clinical parameters

Among responders, bivariate analysis demonstrated a 17.9 ng/mL higher week 24 serum 25(OH)D level among current efavirenz users (*p* = 0.01), whereas current PI use was associated with a 16.2 ng/mL lower week 24 serum 25(OH) D level (*p* = 0.03). After adjusting for age, sex, race, nadir CD4+ T lymphocyte count and baseline 25(OH)D, current efavirenz use was associated with a 21.1 ng/mL increase in week 24 serum 25(OH)D level (*p* = 0.007, Table 2B).

For the effect of duration of ART exposure, after adjusting for sex, age, nadir CD4+ T lymphocyte count and baseline 25(OH)D, per year use of zidovudine was associated with a 7.1 ng/mL lower week 24 serum 25(OH)D (*p* = 0.05). For the other ART drugs/classes, per year exposure was associated with lower week 24 serum 25(OH)D levels (tenofovir = −1.55, *p* = 0.33; efavirenz = −1.36, *p* = 0.60; PI = −0.17, *p* = 0.96), although these did not achieve statistical significance.

Among participants who received vitamin D supplementation, there was no significant difference between median baseline CD4+ T lymphocyte count for responders and non-responders (689 vs. 540 cells/mm^3^, *p* = 0.69). Compared to baseline, week 24 CD4+ T lymphocyte counts increased in both groups (median at week 24 712 cells/mm^3^ for responders vs. 656 cells/mm^3^ for non-responders) (Table 3). Additionally, a significant positive correlation was observed between 24-week changes in CD4+ T lymphocyte counts and 25(OH)D levels among responders (*r* = 0.44, *p* = 0.01). After adjusting for age, sex, race and nadir CD4+ T lymphocyte count, each 1.0 ng/mL increase in 25(OH)D during repletion therapy was associated with a 3.3 cell/mm^3^ increase in CD4+ T lymphocyte count (*p* = 0.06, Table 4).

**Table 4:** Linear models for 24-week CD4+ T lymphocyte count change among participants that achieved vitamin D sufficiency (*n* = 31)

	Unadjusted model (CI)	Adjusted model (CI)
Female sex	−13.02 (−152.29, 126.25)	−57.81 (−186.60, 70.99)
Age (years)	**8.82 (3.04, 14.60)**	**9.42 (3.67, 15.48)**
Non-white race/ethnicity	−38.46 (−175.34, 98.42)	−46.00 (−177.50, 85.50)
Nadir CD^4+^ T lymphocyte (per 100 cells/mm^3^)	0.32 (−71.24, 71.88)	−8.04 (−73.65, 57.56)
Change in 25(OH)D levels (ng/ml)	**4.39 (1.01, 7.76)**	3.25 (−0.05, 6.55)

*25(OH)D* 25-hydroxyvitamin D

Bold font implies statistically significant results assuming a 5% significance threshold

## Discussion

Despite high rates of reported vitamin D insufficiency in treated HIV infection, little has been published on the relationships between ART use and success or failure of vitamin D supplementation. In this cohort of HIV-infected adults on suppressive ART, we demonstrated high rates of vitamin D insufficiency but success of vitamin D repletion with a standardized regimen similar to repletion success rates reported among HIV-uninfected persons [5].

25(OH)D levels have recently been shown to decline in the 24 weeks following initiation of efavirenz with either lamivudine/zidovudine or emtricitabine/tenofovir, but to stabilize thereafter [28]. With an average time on ART of five years, we were not able to assess changes in 25(OH)D levels related to ART initiation; however, vitamin D insufficiency has also been associated with current efavirenz and nevirapine use irrespective of length of treatment [29–33]. While efavirenz use was more common among participants with baseline vitamin D insufficiency in our cohort, we observed a positive association between duration of efavirenz use and success of vitamin D repletion that is consistent with other studies that did not show an inhibitory effect of efavirenz on success of vitamin D repletion [34]. While elucidating a mechanism for this finding is beyond the scope of this study, it is possible that complex interactions between vitamin D, efavirenz and the cytochrome P450 system [14, 35] allow for enhanced efficacy of supplementation.

Despite potentially enhancing vitamin D metabolism, PI therapy has not consistently been associated with vitamin D insufficiency, and PI monotherapy has been associated with decreased risk of vitamin D insufficiency [36]. At baseline, we observed similar rates of PI use among participants with sufficient and insufficient 25(OH)D levels. Additionally, while we observed a strong correlation between PI use and success of vitamin D repletion, PI use was not associated with success or failure of vitamin D repletion in this analysis after adjusting for confounding factors.

In our analysis, baseline rates of bone disease (osteopenia or osteoporosis) did not vary by 25(OH)D level. In an indigenous, adult Brazilian population, lower 25(OH)D levels also did not predict decreased bone mineral density [37], but lower high-density lipoprotein cholesterol were associated with lower 25(OH)D levels. Given the known interactions of high-density lipoprotein with osteoclasts and osteoblasts [38], we assessed this relationship in our cohort, but did not find an association between high-density lipoprotein cholesterol and bone mineral density in the subset of participants who underwent DXA scanning (data not shown).

Lastly, we observed a positive association between changes in 25(OH)D levels and CD^4+^ T lymphocyte counts, a finding that has been previously reported [16, 39–41], although not with consistent results [42–44]. Vitamin D supplementation has also been associated with a decreased Th17-to-Tregulatory lymphocyte ratio [43], decreased expression of CD38+ and Ki67+ on memory CD8+ T lymphocytes [45], increased regulatory T lymphocyte numbers [43, 46], enhanced Th2-dependent cytokine expression and decreased Th1-dependent cytokine levels [47], which may help to limit ongoing immune activation in settings such as treated HIV infection. As such, larger studies designed to look at the effects of vitamin D supplementation on T lymphocyte recovery and immune activation are warranted.

This study has several limitations. First, the sample size was based on the primary objective (to assess the effectiveness of a standardized vitamin D supplementation regimen), and was likely too small to fully evaluate relationships between changes in 25(OH)D levels and specific clinical and demographic factors, including specific ART agents and metabolic parameters. Second, the metabolic and immunological parameters we evaluated were restricted to those routinely performed in accordance with Brazilian guidelines. Third, DXA results were available for only a subset of consenting participants, and therefore do not represent a random sample of our larger cohort. Similarly, the findings presented here cannot be generalized to the entire IPEC cohort or Brazilian HIV-infected population for many reasons, including differences among individuals who were enrolled in the cohort over time, possible cohort effects and the non-probabilistic characteristics of the sample included in this study. Finally, we could not stringently assess adherence to vitamin D supplementation due to supplement formulation.

In conclusion, standardized vitamin D_3_ supplementation was effective in repleting 25(OH)D levels after 24 weeks. Time on efavirenz was positively associated with post-repletion 25(OH)D levels, whereas no significant associations were observed for other antiretroviral agents. The strong correlation and association between increases in 25(OH)D levels and CD4+ T lymphocyte counts supports a benefit of vitamin D supplementation on immunologic recovery, which is particularly relevant to the HIV-infected population.

## Additional file

Additional file 1: Table S1. Comparison between DXA categories at baseline (defined based on Z-score values). (DOCX 15 kb)
