# Supplementary material for: Vitamin D3 supplementation in HIV infection: effectiveness and associations with antiretroviral therapy
Source: Nutr J. 2015 Aug 18;14:81. doi: 10.1186/s12937-015-0072-6 (PMC4538921; doi:10.1186/s12937-015-0072-6)
Supplement: Additional file 1: Table S1. — Comparison between DXA categories at baseline (defined based on Z-score values). (DOCX 15 kb) [file 12937_2015_72_MOESM1_ESM.docx]

Additional file 1: Table S1. Comparison between DXA categories at baseline (defined based on Z-score values).

|  | Normal | Any bone disease* | Total | P-value |
| --- | --- | --- | --- | --- |
|  | (n=16) | (n=38) | (n= 54) |  |
| Male sex | 81% (13) | 66% (25) | 70% (38) | 0.34 |
| Age (years) | 48 (42, 55) | 47 (38, 53) | 47 (39, 53) | 0.25 |
| Non-white race/ethnicity | 38% (6) | 58% (22) | 52% (28) | 0.28 |
| BMI (kg/m^2^) | 26 (24, 27) | 25 (23, 27) | 25 (23, 27) | 0.54 |
| <18.5 | 0% (0) | 3% (1) | 2% (1) |  |
| 18.5-24.9 | 44% (7) | 53% (20) | 50% (27) |  |
| 25-29.9 | 44% (7) | 34% (13) | 37% (20) |  |
| ≥30.0 | 12% (2) | 11% (4) | 11% (6) |  |
| 25(OH)D (ng/mL) | 27 (16, 36) | 24 (18, 35) | 25 (17, 35) | 0.93 |
| Nadir CD4+ T lymphocyte (cells/mm³) | 167 (117, 212) | 172(52, 208) | 170 (77, 210) | 0.32 |
| ≥350 | 0% (0) | 3% (1) | 2% (1) |  |
| 200-349 | 31% (5) | 31% (11) | 31% (16) |  |
| 50-199 | 69% (11) | 42% (15) | 50% (26) |  |
| <50 | 0% (0) | 25% (9) | 17% (9) |  |
| Time on cART (years) | 5 (5, 5) | 5 (4, 5) | 5 (4, 5) | 0.95 |
| Time on TDF (years) | 3 (1, 5) | 3 (1, 5) | 3 (1, 5) | 0.95 |
| Time on AZT (years) | 0 (0, 4) | 0 (0, 4) | 0 (0, 4) | 0.34 |
| Time on EFV (years) | 4 (0, 5) | 4 (2, 5) | 4 (1, 5) | 0.69 |
| Time on PI (years) | 1 (0, 4) | 0 (0, 2) | 0 (0, 3) | 0.85 |
| Current TDF use | 75% (12) | 74% (28) | 74% (40) | 1 |
| Current AZT use | 38% (6) | 26% (10) | 30% (16) | 0.52 |
| Current EFV use | 63% (10) | 79% (30) | 74% (40) | 0.31 |
| Current PI use | 31% (5) | 21% (8) | 24% (13) | 0.96 |

Median (interquartile range) or percent (n) presented

DXA: Dual-energy X-ray absorptiometry; 25(OH)D: 25-hydroxyvitamin D; TDF: tenofovir; AZT: zidovudine; EFV: efavirenz; PI: protease inhibitors; BMI: body mass index.

*Defined as Z score ≤ -1.0
